# Bimaxillary Dentoalveolar Protrusion Case Treated with Anchorage by Buccally Implemented Mini-Implants Using a 3D-Printed Surgical Guide

**DOI:** 10.3390/children10050879

**Published:** 2023-05-14

**Authors:** Georgios Vasoglou, Athanasia Patatou, Michail Vasoglou

**Affiliations:** 1 Private Orthodontic Practice, 17676 Athens, Greece; 2 Department of Orthodontics, School of Dentistry, National and Kapodistrian University of Athens, 11527 Athens, Greece

**Keywords:** bimaxillary protrusion, mini-implants, surgical guide, CBCT, 3D printing

## Abstract

The article presents a case of bimaxillary dentoalveolar protrusion treated by distalizing the upper and lower teeth, using anchorage from mini implants. A 16-year-old male patient presented with severe upper and lower incisor proclination with protruding lips and a convex profile, with a background of bimaxillary dentoalveolar protrusion. Instead of having four premolars extracted, retraction of the dentition was decided with absolute anchorage, provided by mini implants. In order to carry out the procedure in one stage, four mini-implants were inserted as close to the root of the 1st molars as possible. Implementation was facilitated by a surgical template which was created on a digital model and then 3D printed. Accurate placement was achieved and the case was successfully treated by significant uprighting of the incisors and retraction of the anterior dentition, closing the spaces in the upper and lower arch. Facial aesthetics were also improved. A digitally designed surgical guide was utilized in this case of bimaxillary dentoalveolar protrusion in order to facilitate the accurate placement of the mini implants which were used for a one-stage retraction of the dentition.

## 1. Introduction

Bimaxillary protrusion is a pathologic modality which presents dentoalveolar proclination of the upper and lower anterior teeth [1]. Reduced interincisal angle, protruding lips and convex profile are components of this deformity [2]. A Class I skeletal pattern is often identified but skeletal base discrepancies may be recorded. On an ethnic basis, bimaxillary protrusion is mostly found among East Asian and African populations [1,3]. Patients usually seek treatment mainly due to aesthetic reasons in order to gain a better facial profile.

Possible treatment approaches include the extraction of four first premolars and orthodontic retraction of anterior dentition [4] with absolute anchorage, or the surgical repositioning of the anterior segments [5,6,7]. Root resorption and control of anterior segments are matters of controversy between the two approaches [8,9]. Subapical osteotomy was introduced in 1921 [10] and several modifications have been suggested [11,12] due to possible pathologic conditions that may occur as a result of this technique. Some of them are necrosis of the repositioned segments, pulp devascularization of front teeth and alar base widening. These conditions have led to several modifications. Anterior subapical osteotomies are reported to be more efficient when dealing not only with dentition protrusion but with skeletal base discrepancies as well [13].

Retraction of the whole upper and lower dentition is now feasible with the use of zygomatic arch anchorage [14,15], palatal plates [16,17,18], or mini-implants [19]. The use of mini-implants simplify the procedures and biomechanics in mechanotherapy. Extra-alveolar placement of stainless-steel orthodontic implants with large dimensions (length and diameter) have been used by Chang et al. [20,21] in the infrazygomatic crest area or in the mandibular buccal shelf for a single-stage retraction of the entire dentition. Other sites for mini-implant placement in order to gain space for greater retractions are between molars or distal to the second molar in the mandible [22,23]. In more severe cases of bimaxillary protrusion, two-stage retraction, with the use of temporary anchorage devices (TADs), can be utilized by changing the position of the TADs before the root of the premolar or molar contacts the TAD.

Utilizing CBCT imaging [24] and intraoral scanning for diagnostic reasons [25] in conjunction with 3D printing have made possible the fabrication of surgical guides for accurate mini-implant placement [26,27]. Three-dimensional printing is an additive technology, i.e., a layer-by-layer manufacturing process. In dentistry, 3D printing is used for manufacturing surgical templates, restorations (crowns, inlays, bridges, dentures) and orthodontic appliances [28,29,30]. There are several 3D printing technologies: stereolithography (SLA), Direct Light Processing (DLP), Liquid Crystal Display (LCD), Fused Filament Fabrication (FFF) and Polyjet Photopolymer technology (PPP) [31].

This case report describes a one-stage retraction of the dentition with anchorage of orthodontic implants, in a patient presenting a moderate bimaxillary dentoalveolar protrusion. The procedure was facilitated by a computer designed guide for accurate placement of the implants.

## 2. Materials and Methods

**Figure 1:**
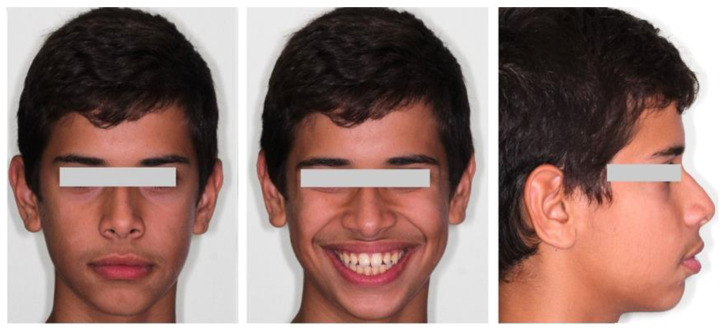
Pretreatment facial photographs.

**Figure 2:**
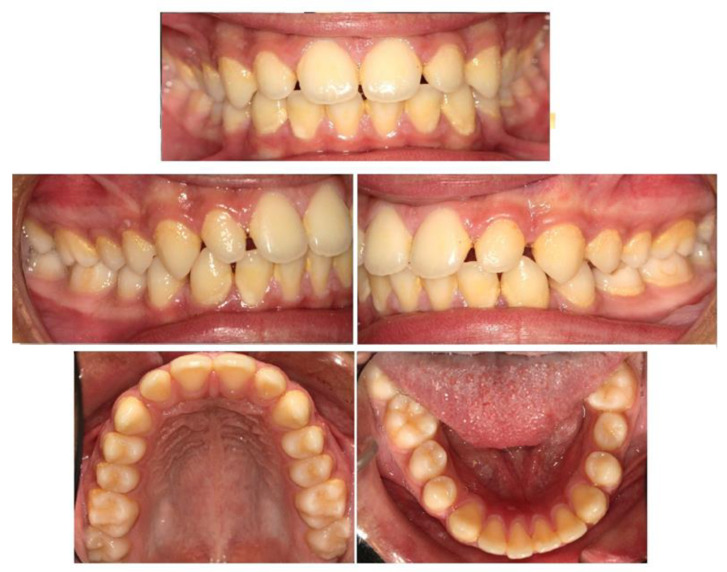
Pretreatment intraoral photographs.

A 16-year-old male patient presented in a private orthodontic clinic in Athens, Greece, with a symmetric face but convex profile, a reduced labionasal angle and protruding lips (Figure 1) seeking orthodontic therapy. A Class I canine and molar relationship, a slightly increased overjet and a normal overbite were recorded (Figure 2). His dental and medical history were free from pathologic findings. There was a mild spacing in the maxillary and mandibular dental arch in the anterior region and a moderate curve of Spee. The dental midlines coincided with the facial midline. The third molars, which were developing at that time, presented relatively favorable inclination and enough space for eruption.

**Figure 3:**
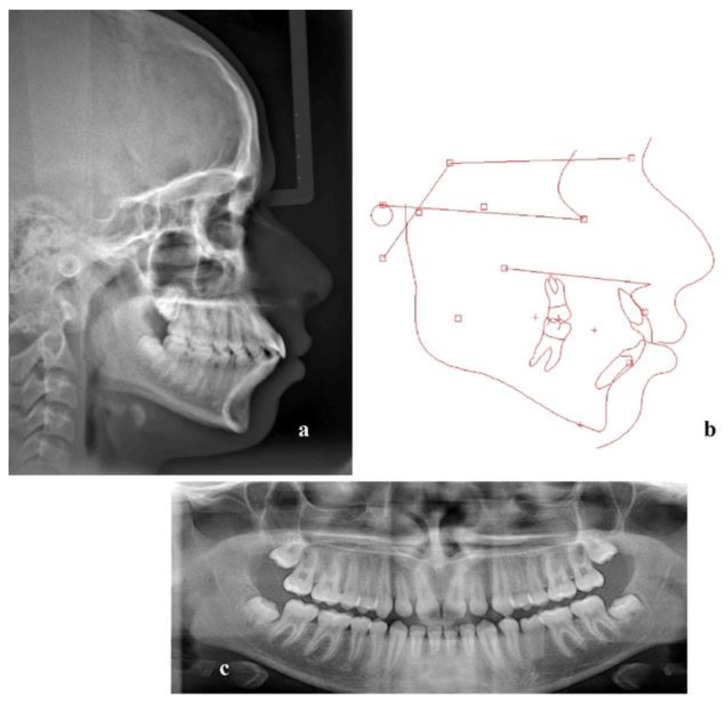
(**a**) Lateral cephalogram, (**b**) tracing and (**c**) panoramic X-ray before treatment.

A lateral cephalogram and a panoramic X-ray (Figure 3) were acquired and the lateral cephalometric tracing and measurements indicated a moderate Class II skeletal pattern (ANB: 5.1°; Wits appraisal: 0.1 mm) with a slightly increased mandibular plane angle (Go-Gn/SN: 27,1°). The incisors in the upper and lower jaw were severely proclined (U1/PP: 125.9°; L1/MP: 110.8°) and the interincisal angle (104.6°) was reduced (Table 1). The diagnosis was bimaxillary dentoalveolar protrusion.

The treatment goals for the patient were uprighting of the proclined incisors in the upper and lower jaw, closing of the spaces in the upper and lower arch and establishing a normal overbite and overjet. The facial profile would be improved by reducing the lip prominence.

The upper and lower dentition were scheduled to be retracted with the use of anchorage from mini-implants, instead of having four premolars extracted. It was decided that mini-implants should be implemented between the first molar and second premolar on both arches, but as near to the first molars as periodontal conditions would permit, so as to avoid a two-stage procedure. The treatment plan was part of a study protocol which was approved by the Scientific and Ethics Committee of 401 Military Hospital in Athens, Greece (ref: No 10/8-12-2020). The patient and his parents were informed about the treatment procedure and they signed a consent form.

**Figure 4:**
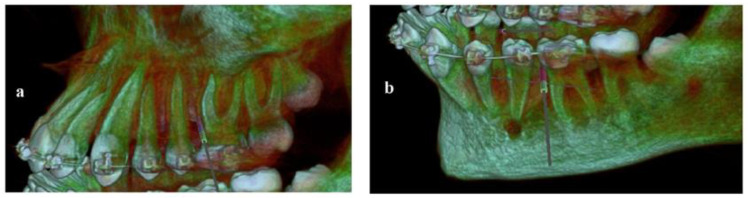
Virtual mini-implants placed on the digital model. (**a**) Upper jaw; (**b**) lower jaw.

In order to ensure the exact placement of the mini implants, a surgical guide was designed and manufactured. Fixed appliances were inserted on both arches and after leveling and aligning stages and when rectangular (0.019 × 0.025-inch) stainless steel archwires were in place, a CBCT imaging of the upper and lower jaw was acquired (Planmeca ProMax^®^ CBCT system, Planmeca Oy, Helsinki, Finland,90 kVp/4–10 mA, 200–400 μm voxel size). Additionally, an intraoral scan was performed (CS 3600, oral scanner, Carestream Dental, LLC, Atlanta, GA, USA). A stereolithography (.stl) file was acquired and was uploaded into the Blue Sky Plan software (Version 4.9.4, Blue Sky Bio, LLC, Libertyville, IL, USA). Digital Imaging and Communications in Medicine (DICOM) files were also uploaded and combined with the .stl file with several matching points and a digital model was created. Virtual mini-implants were created with the software (identical to length and diameter of the real ones) and inserted at the planned position (Figure 4).

**Figure 5:**
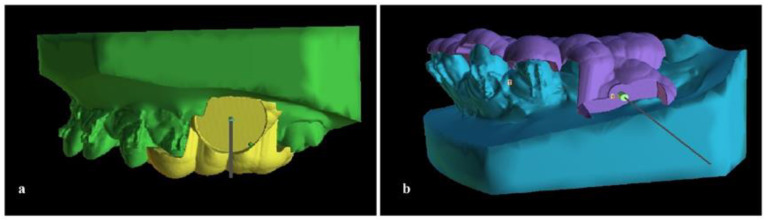
The surgical guide on the digital model. (**a**) Upper jaw; (**b**) lower jaw.

On the digital model, two surgical guides (one for the upper and one for the lower arch) were designed leaning on the occlusal surfaces of the premolars and molars, incorporating projections with holes for guiding the mini-implants. The thickness of the guides was 2 mm. The diameter of the guiding holes was set to 1.3–1.5 mm while the length was designed to be 3 mm, in order to provide adequate guidance (Figure 5).

**Figure 6:**
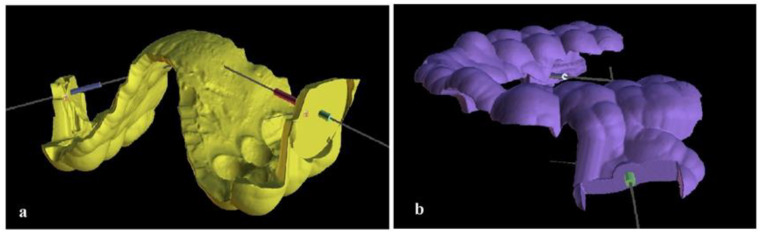
The .stl models of the surgical guides. (**a**) Upper jaw guide; (**b**) lower jaw guide.

The .stl files of the surgical guides (Figure 6) were then printed in a biocompatible resin using a 3D printer (Formlabs Form 2).

**Figure 7:**
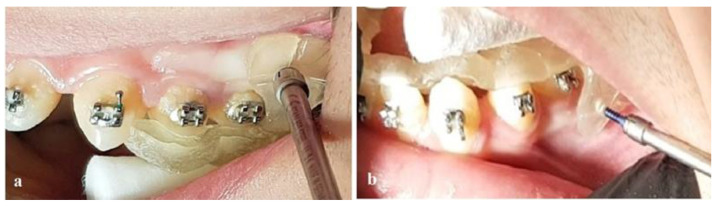
Implementation of mini-implants utilizing the 3D-printed surgical guides. (**a**) Upper jaw; (**b**) lower jaw.

The guides were tested for proper contact on teeth, with no interference from tubes and brackets and necessary grinding was performed. After local anesthesia (3% solution of mepivacaine), two mini-implants for the upper jaw (Aarhus, Medicon eG Ø: 1.5 mm) and two mini-implants for the lower jaw (Abso Anchor, Dentos Inc., Daegu, Republic of Korea; Ø: 1.3 mm) were placed with the help of the guides into the planned positions (Figure 7).

**Figure 8:**
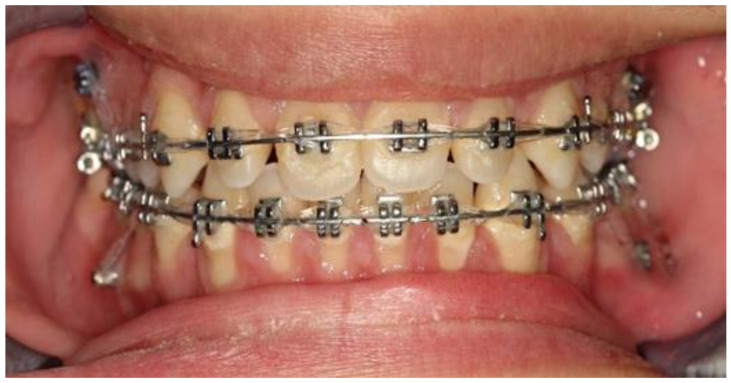
Retraction of upper and lower dentition using elastic chains and anchorage from the mini-implants.

One week after implementation, retraction of the teeth using elastic chains, mounted on the mini-implants’ neck and canine hooks, was carried out (Figure 8).

**Figure 9:**
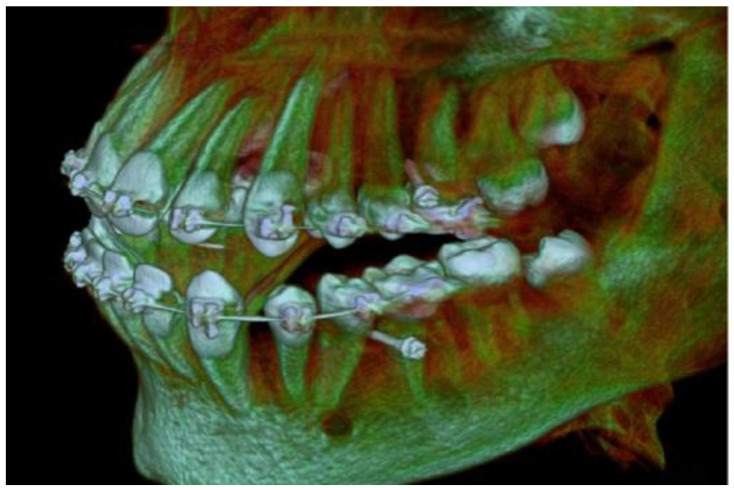
CBCT imaging during retraction with mini-implants in place.

The visiting intervals were set to four weeks and the whole process lasted about nine months. In the sixth month and before completing the retraction, a new CBCT was obtained in order to check the mini-implants’ position and the integrity of the roots of the premolars (Figure 9).

After finishing procedures, the mini-implants were removed and after completion of the treatment essix retainers were provided.

## 3. Results

**Figure 10:**
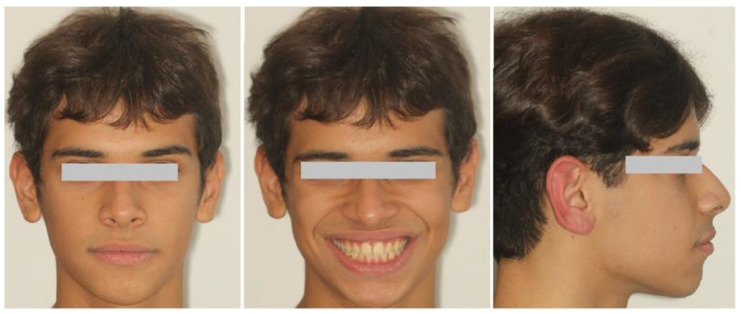
Posttreatment facial photographs of the patient.

**Figure 11:**
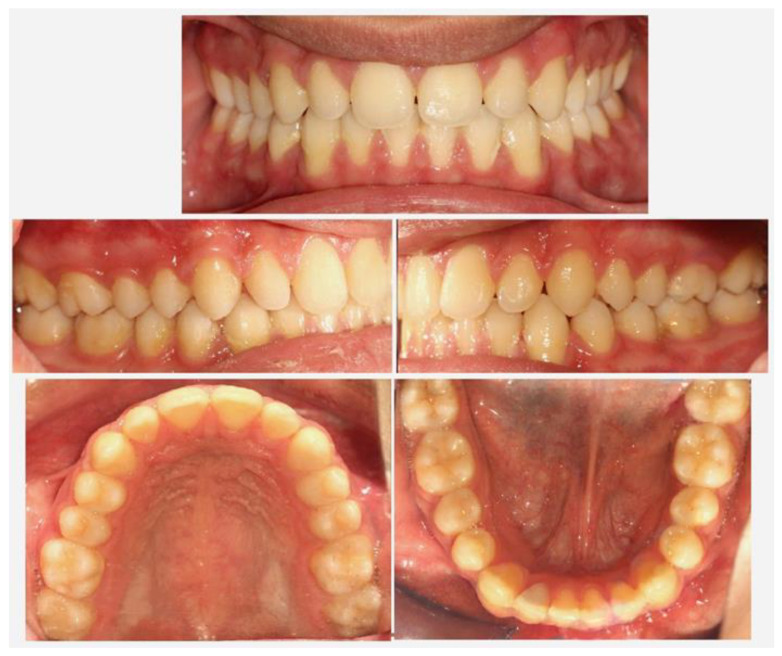
Posttreatment intraoral photographs of the patient.

**Figure 12:**
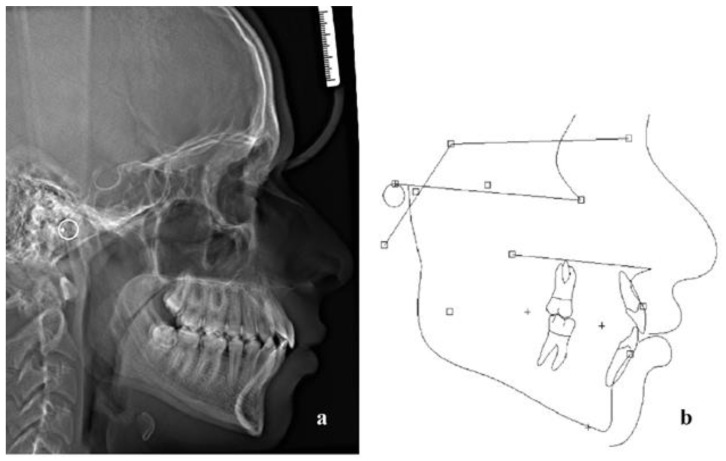
(**a**) Posttreatment lateral cephalogram and (**b**) tracing.

**Figure 13:**
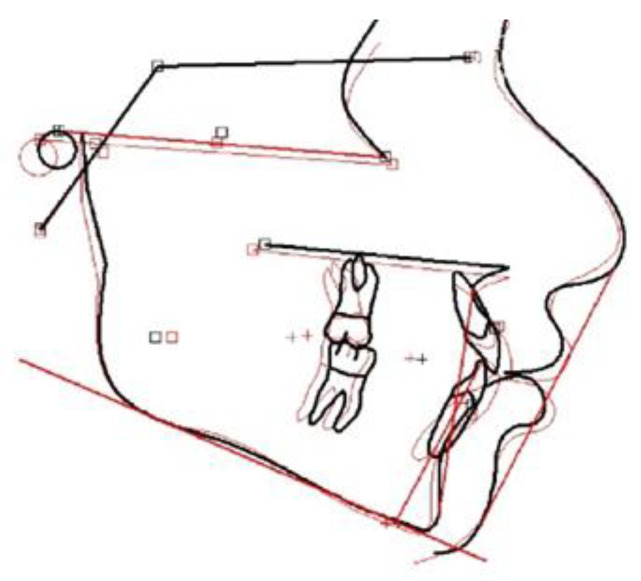
Superimposition of pre and posttreatment tracings on the anterior cranial base (red line: pretreatment, black line: posttreatment).

The treatment objectives, as indicated by the posttreatment records, were achieved (Table 1), as a significant amount of retraction of the anterior dentition was recorded. The retraction, in addition to the closing of the spaces in the anterior region, led to uprighting of the proclined upper and lower anterior teeth and to establishing normal dental relationships. As indicated by the data presented in Table 1, the skeletal pattern (1–5 measurements) was not significantly affected by the treatment procedures, while the dental and aesthetic outcomes (6–9 measurements) were positively affected. Inclination of the upper and lower incisors was improved; the facial aesthetics and profile were also improved and Class I relationships were maintained (Figure 10 and Figure 11). A posttreatment lateral cephalogram (Figure 12) and superimposition of the tracings (Figure 13) indicated the improvement of the facial profile and in dental relationships, mainly in the anterior region. 

## 4. Discussion

Since improvement of facial aesthetics is often the main demand of patients presenting with bimaxillary protrusion, a severe case with a skeletal background might favored, in terms of profile changes, a surgical approach [32]. On the other hand, an orthodontic treatment approach with four premolar extractions, in less severe cases with dentoalveolar background and proclined anterior teeth, is efficient, though there are certain limitations such as prolonged treatment time, periodontal problems on closing extraction spaces and compromised social factors and thus the subapical osteotomy technique might be preferable [6]. 

Twenty years ago, orthodontic microscrews and miniplates were introduced as means to enhance anchorage [33,34,35]. With the introduction of such devises in treatment planning, the need for patient cooperation that was mandatory regarding conventional devices, was reduced. Total distalization of the whole upper and lower dentition became feasible [14,15,16,17,18,19] as a treatment alternative in moderate dentoalveolar protrusion cases [36]. In the case presented, there were some spaces between teeth, especially in the lower arch were some additional space was gained after derotation of the second left lower premolar. Therefore, there was no need for distalization of the molars which is the objective in cases with crowding and proclination of anterior teeth. Uprighting of the anterior upper and lower teeth and space closure and utilizing absolute anchorage with the help of mini-implants was enough to solve the case and deal with the convex profile. Upper molar distalization, when needed, can be achieved by conventional appliances like headgear [37] or the pentulum [38], but there are certain limitations such as patient compliance, anchorage loss in frontal areas of the dental arch and tipping of molars instead of bodily movement. On the other hand, distalization of the whole dentition in the lower arch is difficult and the anchorage from mini-implants made this approach feasible. In order not to interfere with the roots of the distalizing teeth and complete the task in one stage, the placement of palatal plates [19] or mini-implants to the infrazygomatic crest [20] in the upper jaw and implementation of TADs in the retromolar area for the lower arch [39], has been suggested.

When interradicular areas are used for mini-implant placement, such as between molars or second premolars and first molars, a two-stage procedure should be probably followed and mini-implants must be replanted in order not to come in contact with the distalizing teeth. The alternative is to insert the mini-implants as close to the root of the first or second molar as possible and distalize the dentition, provided that the interradicular space is enough and the anatomic features, like the root shape, are favorable. In this case, the radiographic findings encouraged the later prospective and the use of a surgical template was designed for accuracy in mini-implant placement. Several methods have been proposed in the literature for this task: three-dimensional stents for exact placement [40], stainless-steel tubes incorporated into 3-dimensional templates [41] or fabrication of guides using acrylic resin on patient plaster models [42,43]. However, the use of CBCT imaging [44], CAD/CAM technology and 3D printing [45] led to more accurate results. Surgical guides can now be manufactured after acquiring a CBCT, performing an intraoral scan and combining these data into a digital model [26]. Moreover, mini-implant placement, using guides designed and manufactured with digital technology, have been introduced for the exposure and traction of impacted canines [46]. The method that was used in the case presented was introduced in previous research [27]. Virtual mini-implants were placed on a digital model of a patient’s mouth at the exact place near to the mesial root of the first molars. The guide that was designed and then 3D printed, incorporated a projection with a guiding hole for the implant, which was broken under screwing pressure and removed. As a result, the mini-implants were placed leaving enough space for teeth retraction in one stage. The specific procedure ensured some critical factors for mini-implant success, like the point of insertion in the attached gingiva and the inclination of the mini-implant to the occlusal plane. In the case that crowding existed (unlike in the case presented), the retraction of upper molars after third molar extraction should be performed.

The importance of the CBCT imaging in dentistry is now recognized and accepted [24].

In orthodontic cases, CBCT imaging is not routinely indicated for diagnosis. However, it is valuable in the assessment of impacted and ectopic teeth [47,48] and in diagnosing root damage of the lateral incisors due to canine impaction [49]. In assessment of skeletal abnormalities like asymmetries and clefts, CBCT can be effectively used for treatment planning [50]. Additionally, CBCT can be utilized for 3-dimensional airway analysis in cases of obstructive sleep apnea [51]. However, there is a consideration regarding deleterious effects of the amount of ionizing radiation. This can be overcome by utilizing a limited field of view (FOV) of the jaws and a low dose mode. Thus, in similar cases, clinicians should evaluate the need for adopting digital procedures in diagnosis and mini-implants placement, instead of utilizing conventional 2D imaging and a two-stage strategy.

In this case, the use of a guide designed and created by digital technologies helped in achieving the exact implementation of the orthodontic mini-implants which in turn led to successful retraction of the teeth. 

## 5. Conclusions

Bimaxillary dentoalveolar protrusion can be successfully treated with anchorage from mini-implants for retraction of the teeth. Additionally, the use of a 3D-printed surgical guide can facilitate the exact placement of the mini-implants in a one-stage procedure.

## Figures and Tables

**Table 1:** Skeletal and dental measurements pre- and post-treatment in lateral cephalograms.

Measurement	Mean ± SD	Pretreatment	Posttreatment
1. SNA (°)	81 ± 3	88.1	89
2. SNB (°)	78 ± 3	83	84.2
3. ANB (°)	3 ± 2	5.1	4.9
4. Wits (mm)	1 ± 2.9	0.1	0.8
5. GoGN/SN (°)	32.5 ± 5.2	27.1	25.8
6. U1/PP (°)	109 ± 6	125.9	117.1
7. L1/MP (°)	93 ± 6	110.8	94.5
8. Interincisal angle (°)	135 ± 10	104.6	130.2
9. Labionasal angle (°)	95.96 ± 2.57	106.6	99

## Data Availability

Data available upon reasonable request.
